# Portopulmonary hypertension and the risk of high right ventricular systolic pressure in liver transplant candidates

**DOI:** 10.1371/journal.pone.0267125

**Published:** 2022-04-19

**Authors:** Ryoko Hayashi, Tomomi Kogiso, Noriko Kikuchi, Kana Yamamoto, Shinichi Nakamura, Hiroto Egawa, Nobuhisa Hagiwara, Katsutoshi Tokushige

**Affiliations:** 1 Department of Internal Medicine, Institute of Gastroenterology, Tokyo Women’s Medical University, Shinjuku-ku, Tokyo, Japan; 2 Department of Cardiology, Tokyo Women’s Medical University, Shinjuku-ku, Tokyo, Japan; 3 Department of surgery, Institute of Gastroenterology, Tokyo Women’s Medical University, Shinjuku-ku, Tokyo, Japan; Vanderbilt University Medical Center, UNITED STATES

## Abstract

**Aim:**

Portopulmonary hypertension (PoPH) is a rare and serious complication of liver cirrhosis and portal hypertension that can interfere with liver transplantation (LT). We evaluated the prevalence of PoPH and the clinical features of right ventricular systolic pressure (RVSP), which is equivalent to pulmonary artery systolic pressure, in LT candidates.

**Methods:**

This was a single-center retrospective study. A total of 157 Japanese patients with decompensated liver cirrhosis or portal hypertension (76 men, median age = 52 years [range: 18–68 years]) were enrolled. The relationships between RVSP and clinical parameters, and the prevalence of PoPH in LT candidates, were evaluated.

**Results:**

The cardiological parameters were as follows: brain natriuretic peptide (BNP), 39.1 (4.0–780.5) pg/mL; RVSP, 31.2 (16.0–122.4) mmHg; ejection fraction, 58% (28–72%); and mean peak tricuspid regurgitation velocity, 2.3 (1.5–5.3) m/s. The RVSP was significantly higher in females (*p* = 0.02) and primary biliary cholangitis (PBC) patients (*p* = 0.01), and was weakly correlated with the BNP level (r = 0.40, *p* = 0.01). For RVSPs of < 36 and ≥ 36 mmHg, the 5-year survival rates were 36.1% *versus* 34.1%, and 85.4% *versus* 85.3%, in non-LT and LT cases, respectively (*p* = 0.47 and 0.69, respectively). Among six patients with an RVSP ≥ 50 mmHg, three (1.9%) were diagnosed with PoPH and treated with vasodilators.

**Conclusions:**

PoPH was observed in 3 cases (1.9%) in 157 LT candidates. In patients with suspected mild pulmonary hypertension (RVSP, 36 - 50 mmHg), LT was successfully performed.

## Introduction

Portopulmonary hypertension (PoPH) is an uncommon complication of cirrhosis and portal hypertension diagnosed based on concomitant occurrence of pulmonary arterial hypertension (PAH) [1–3]. PoPH was found in 5–15% of PAH cases [4], and 1–6% of patients with portal hypertension have PAH [3, 5–7]. The prevalence of PoPH is 2–6% in liver cirrhosis and liver transplantation (LT) candidates [5–7]. PoPH is defined as a mean pulmonary artery pressure (mPAP) of > 25 mmHg, pulmonary vascular resistance (PVR) of > 120 dyn× s/cm^-5^, and pulmonary capillary wedged pressure (PCWP) of < 15 mmHg in the presence of portal hypertension [8–10]. Other definitions include a PVR of > 2 or 3 Wood units, as measured by right heart catheterization [10, 11].

Echocardiography is useful for detecting PoPH [12]. In healthy control subjects, the normal right ventricular systolic pressure (RVSP) is reportedly 28 ± 5 mmHg (range 15–57 mmHg). According to these data, mild pulmonary hypertension can be defined as a pulmonary artery systolic pressure (PASP) of approximately 36–50 mmHg, equivalent to an RVSP of approximately 36–50 mmHg [13, 14]. The clinical signs and symptoms of pulmonary hypertension are pulmonic valve insufficiency, paradoxical septal motion, right ventricular hypertrophy/dilatation, and poor tricuspid annular systolic excursion. Patients with an RVSP of > 50 mmHg according to echocardiography require catheterization [15]. Additionally, in patients with an RVSP ≥ 36 mmHg, when physical findings are consistent with pulmonary hypertension, further evaluation is needed. Colle *et al*. [5] reported no false-negatives using echocardiography for detecting PoPH; however, it had a poor positive predictive value, so right heart catheterization is recommended for diagnosis.

Female sex and autoimmune liver disease are associated with a higher risk of PoPH (odds ratios [ORs], 4 and 9.8, respectively) [16]. In the pathophysiology of PoPH, increased PVR may occur due to pulmonary vasoconstriction, altered levels of circulating mediators, or shear stress, and can lead to classic vascular remodeling [17, 18]. As mediators, endothelin (ET)-1 thromboxane-B1, interleukin-6 and serotonin have been implicated in the pathophysiology of PoPH [19]. Conversely, vasodilators such as nitric oxide (NO) and prostaglandin I2 (prostacyclin) may be decreased in PoPH, facilitating vascular remodeling and the vasoproliferative response.

Because mild PoPH is not associated with mortality after LT, it worsens the overall prognosis of moderate to severe cases [20]. mPAP < 35 mmHg is required for LT [21, 22] and vasodilatory therapy is improving the mortality rate of LT [10, 22–24]. Ashfaq *et al*. [25] reported that 12 of 16 (75%) cases had a reduction of mPAP to < 35 mmHg, and 11 successfully underwent deceased donor LT (DDLT). The 1-year survival rate was excellent, at 91%.

PoPH cases have high mPAP and PVR values; we investigated the survival rates and clinical features of high-RVSP LT candidates, as well as the risk factors. Moreover, the prevalence of PoPH was evaluated in Japan.

## Methods

### Patients and study design

This was a single-center, retrospective observational study conducted between June 2011 and September 2021. A total of 157 Japanese patients with liver cirrhosis or portal hypertension (76 men, 48.4%) considered for LT were enrolled. Cirrhosis or portal hypertension was diagnosed based on imaging and biochemistry results. LT was performed in 88 cases (79 living donor LT [LDLTs] and 9 DDLTs). Of the other 69 cases, 22 were on the waiting list, 14 were not indicated for LT due to malignancy or other complications, and 33 died before LT.

This study was conducted in accordance with the principles of the Declaration of Helsinki and the ethics regulations of Tokyo Women’s Medical University Hospital (TWMU, Tokyo, Japan). The TWMU Institutional Review Board approved the study protocol.

### Clinical parameters

The following baseline characteristics of the patients were assessed: age, sex, clinical history, primary hepatic diseases, and complications of cirrhosis (*i*.*e*., encephalopathy, esophageal/gastric varices, ascites, and hepatocellular carcinoma [HCC]). Blood samples for biochemistry and hematological data were collected at the time of evaluation for LT. Laboratory tests were performed to determine the serum concentrations of albumin, total bilirubin, aspartate aminotransferase, alanine transaminase, creatinine, and brain natriuretic peptide (BNP), the prothrombin time (PT%) and the PT international normalized ratio. The Child–Turcotte–Pugh (CTP) [26] and Model for End-Stage Liver Disease (MELD) scores [27] were calculated and used for evaluating liver function. For estimation of cardiac function, echocardiography was performed. The ejection fraction (EF) and mean peak tricuspid regurgitation (TR) velocity were also measured. RVSP was determined using the modified Bernoulli equation: RVSP (mmHg) = 4 x TR^2^+ right atrial pressure estimate (10 mmHg) [7].

### Diagnosis of PoPH

All patients underwent echocardiography; however, RVSP could not be measured in 20 cases. In cases with an RVSP ≥ 50 or ≥ 36 mmHg and physical findings consistent with pulmonary hypertension, right heart catheterization was performed (n = 3). Pulmonary hypertension was defined as a resting mPAP > 25 mmHg with pulmonary artery wedge pressure < 15 mmHg and PVR > 3 Wood units, as measured by right heart catheterization [10].

### Endoscopic grading of esophageal varices

Endoscopic grading of esophageal varices was based on the guidelines of the Japanese Research Society for Portal Hypertension [28]. Esophageal varix was classified as follows: F1, small straight varix; F2, enlarged tortuous varix occupying less than one-third of the lumen; and F3, large coil-shaped varix occupying more than one-third of the lumen.

### Follow-up and outcomes

The patients were hospitalized and evaluated for LT. After discharge, patients were followed up every 1–2 months at the outpatient clinic, when blood samples were obtained. Prognosis was evaluated in terms of survival time. The observation periods were from the date of LT or hospitalization for LT to death, and the time of censoring (July 2021).

### Statistical analysis

Data are medians with minimum and maximum values. The significance of differences between the RVSP < 36 and ≥ 36 mmHg groups was assessed by the Mann–Whitney U test, χ^2^ test, or Wilcoxon rank sum test using SPSS software (version 25; SPSS Inc., Chicago, IL). Multivariate regression analysis was performed to compare the groups. Factors highly significant in univariate analyses were subjected to multivariate analysis. The following factors were used to estimate ORs and 95% confidence intervals (CIs): sex, presence of varices, BNP, and EF. Mean peak TR velocity was not included due to collinearity. The survival rate was subjected to Kaplan–Meier analysis. Subgroup analysis was performed by stratifying non-LT and LT patients according to RVSP level (< 36 *versus* [vs.] ≥ 36 mmHg). Differences between groups were analyzed by log-rank test and *p* < 0.05 was taken to indicate statistical significance.

## Results

### Baseline characteristics of cirrhotic candidates for LT

The median age of the 157 patients was 52 (range: 18–68) years, and 76 (48.4%) were male (Table 1). The primary liver diseases were viral hepatitis (hepatitis C, 27 cases; hepatitis B, 8 cases), alcoholic liver disease/nonalcoholic fatty liver disease (25/24 cases), autoimmune hepatitis (AIH, 4 cases), primary biliary cholangitis (PBC, 20 cases), primary sclerosing cholangitis (16 cases), biliary atresia (12 cases), Wilson’s disease (3 cases), Budd-Chiari syndrome (4 cases), and others (14 cases, including 1 of extrahepatic portal vein obstruction). With regard to complications of liver cirrhosis, hepatic encephalopathy (36 cases, 22.9%), esophageal/gastric varices (108 cases, 75.0%), portal thrombosis (25 cases, 15.9%), and HCC (25 cases, 15.9%) were all observed.

**Table 1:** Baseline characteristics of the patients.

Variable	Total (*n* = 157)	RVSP <36 mmHg * (*n* = 105)	RVSP ≥36 mmHg * (*n* = 32)	*p-*value*
Age (years)	52 (18–68)	52 (18–67)	54 (20–66)	0.70
No. of males (%)	76 (48.4%)	55 (52.4%)	9 (28.1%)	0.02
Primary liver disease				0.07 **
Viral (HCV/HBV)	27/8	20/8	5/0	
Alcoholic/nonalcoholic	25/24	17/13	5/6	
AIH/PBC/PSC	4/20/16	4/9/12	0/8/2	
Briary atresia	12	10	2	
Wilson’s disease	3	3	0	
Budd-Chiari syndrome	4	3	1	
Others	14	9	4	
Primary PBC/non-PBC	20/137(14.6%)	9/96(9.4%)	8/24(25%)	0.01
Complications (%)				
Hepatic encephalopathy	36 (22.9%)	26 (24.8%)	5 (9.6%)	0.28
Esophageal/gastric varices	108/144 (75.0%)	78/95 (82.1%)	17/30 (56.7%)	<0.01
Hepatocellular carcinoma	25 (15.9%)	19 (18.1%)	2 (6.3%)	0.11
Portal vein thrombus	25 (15.9%)	20 (19.1%)	4 (12.5%)	0.36
**Laboratory data**				
BNP (pg/mL)	39.1 (4.0–780.5)	35.0 (4.0–338.1)	90.8 (6.8–780.5)	<0.01
CTP score	11 (6–14)	11 (7–14)	11 (6–14)	0.12
CTP class A/B/C	2/35/111	0/22/60	2/6/39	
MELD score	17 (3–37)	17 (6–30)	19 (3–37)	0.87
LT (DDLT/LDLT)	88 (9/ 79)	58 (6/52)	19 (3/16)	0.53
**Hemodynamic variables**				
Ejection fraction (%)	58 (28–72)	58 (28–69)	60 (51–72)	0.01
RVSP (mmHg)	31.2 (16.0–122.4)	-	-	-
Peak TR velocity (m/s)	2.3 (1.5–5.3)	2.2 (1.5–2.5)	2.8 (2.6–5.3)	<0.01
LA diameter (cm)	3.8 (2.4–5.3)	3.7 (2.4–5.3)	4.1 (2.7–5.0)	0.36

** Wilcoxon rank sum test

AIH, autoimmune hepatitis; BNP, brain natriuretic peptide; CTP, Child–Turcotte–Pugh; DDLT, deceased donor LT; HBV, hepatitis B virus; HCC, hepatocellular carcinoma; HCV, hepatitis C virus; *n*, number of patients; LA, left atrium; LDLT, living donor LT; LT, liver transplantation; MELD, model for end-stage liver disease; PBC, primary biliary cholangitis; PSC, primary sclerosing cholangitis; RVSP, right ventricular systolic pressure, TR tricuspid regurgitation.

### Cardiac parameters of LT candidates

**Fig 1:**
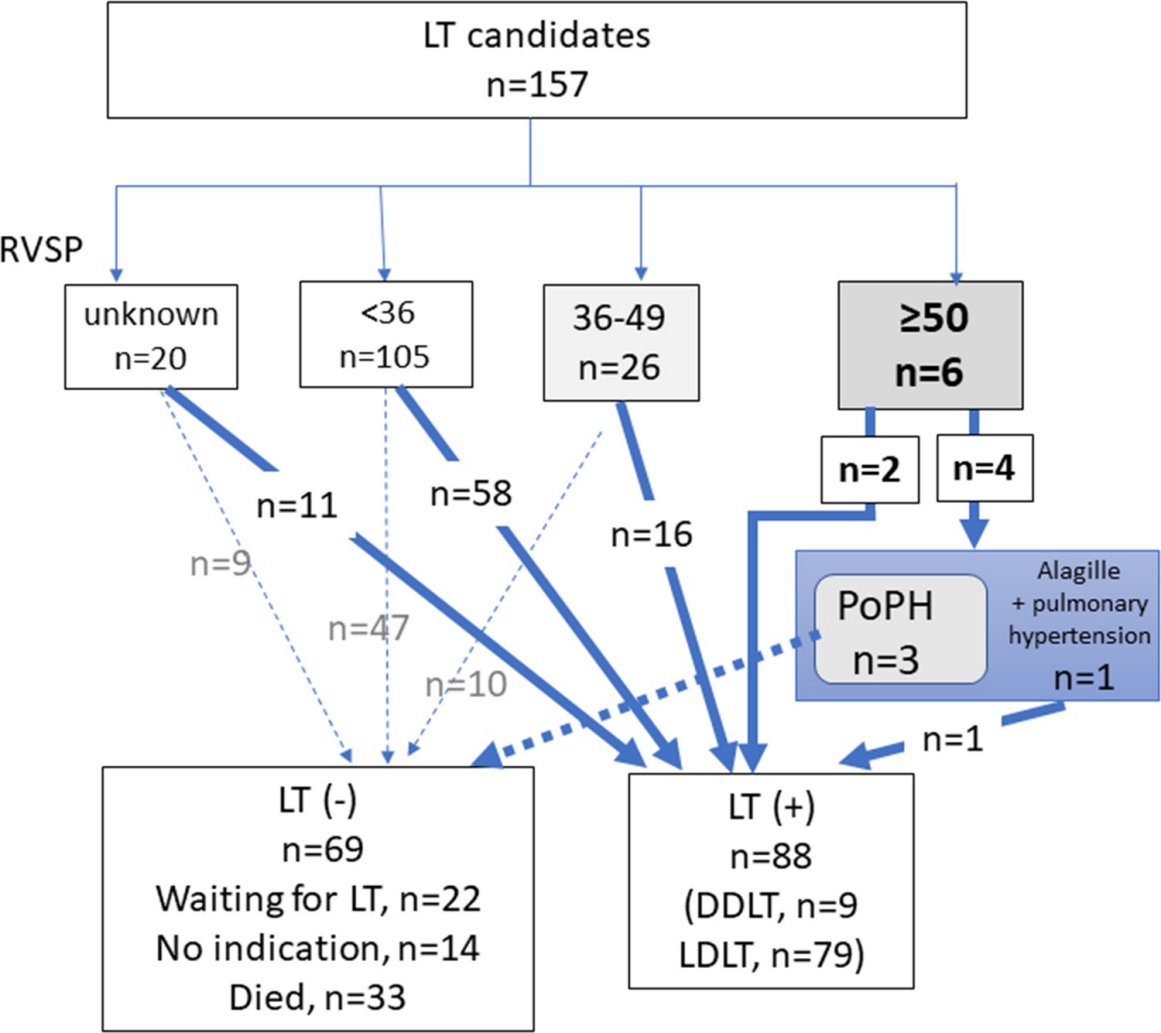
Flow-chart of the LT candidates. Overall, 157 patients underwent LT for end-stage liver disease. According to cardiography, 105, 26, and 6 cases had RVSPs of < 36, 36–49, and ≥ 50 mmHg, respectively. LT was performed in 88 cases (9 cases of DDLT and 79 of LDLT). Three cases (1.9%) were diagnosed with PoPH. LT, liver transplantation; PAH, pulmonary arterial hypertension; PoPH, portopulmonary hypertension.

**Fig 2:**
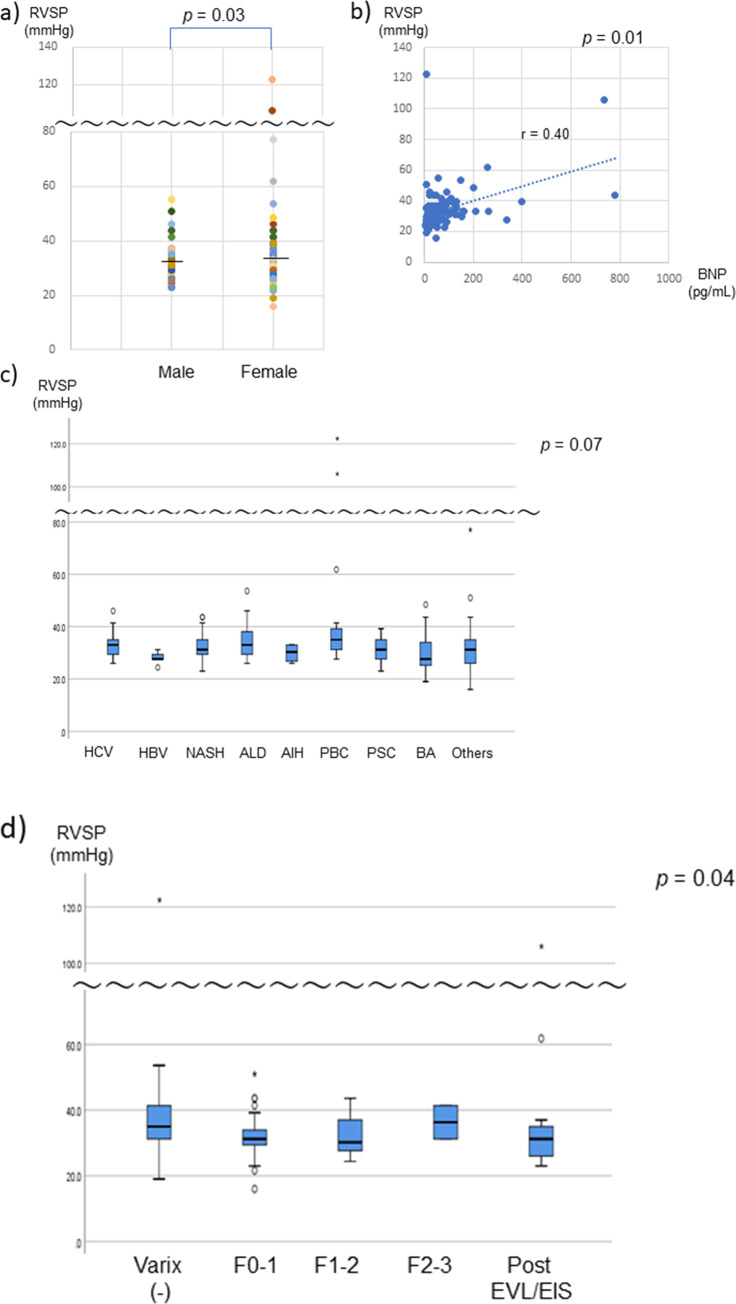
RVSP by sex, BNP, liver disease, and esophageal/gastric varices in LT candidates. (a) Sex, (b) BNP, (c) liver disease, and (d) esophageal/gastric varices. The RVSP was significantly higher in females compared to males (*p* = 0.03) (a). RVSP and the BNP level were weakly correlated (r = 0.40, *p* = 0.01) (b). The RVSP tended to be higher in PBC patients (*p* = 0.07) (c)*. The RVSP was significantly higher in patients without esophageal/gastric varices (*p* = 0.04) (d)*. *Wilcoxon rank sum test. AIH, autoimmune hepatitis; ALD, alcoholic liver disease; BA, biliary atresia; BNP, brain natriuretic peptide; EIS, endoscopic injection sclerotherapy; EVL, endoscopic variceal ligation; HBV, hepatitis B virus; HCV, hepatitis C virus; LT, liver transplantation; NASH, nonalcoholic steatohepatitis; PBC, primary sclerosing cholangitis; PSC, primary sclerosing cholangitis; RVSP, right ventricular systolic pressure.

The cirrhotic patients had a median BNP of 39.1 (4.0–780.5) pg/mL. According to echocardiography, the EF was 58% (28–72%), the mean peak TR velocity was 2.3 (1.5–5.3) m/s, and the RVSP was 31.2 (16–122.4) mmHg. An RVSP of ≥ 50 mmHg was observed in 6 cases, and 36–49 mmHg in 26 cases (Fig 1). The RVSP was significantly higher in females compared to males (Fig 2A). RVSP and BNP were weakly correlated (r = 0.40, *p* = 0.01, Fig 2B). The RVSP tended to be elevated in PBC patients among several background liver diseases (Wilcoxon rank sum test; *p* = 0.07, Fig 2C). The RVSP was higher in patients without esophageal/gastric varices (*p* = 0.04, Fig 2D). (Frequency distribution of each parameter of liver and cardiac function was shown in S1 Fig).

### Comparison of clinical features by RVSP level in LT candidates

Among LT candidates with RVSPs of < 36 and ≥ 36 mmHg, female sex (*p* = 0.02) and the prevalence of PBC were significantly increased (*p* = 0.01), while the cardiac parameters BNP (*p* < 0.01), EF (*p* = 0.01), and mean peak TR velocity (*p* < 0.01) were significantly increased in patients with an RVSP ≥ 36 mmHg (Table 1). Esophageal/gastric varices was negatively associated with an RVSP ≥ 36 mmHg. The results of a multivariate analysis of the risk of RVSP ≥ 36 mmHg are shown in Table 2. The cardiac parameters were positive predictors (EF; OR, 1.23, 95% CI, 1.028–1.471, *p* = 0.02 and BNP; OR, 1.01, 95% CI, 1.003–1.017, *p* = 0 .04) and esophageal/gastric varices was an independent negative predictor of an RVSP ≥ 36 mmHg (OR, 0.21, 95% CI, 0.070–0.611, *p* = 0.04).

**Table 2:** Predictors of an RVSP ≥ 36 mmHg.

Variable	Odds ratio	95% confidence interval	*p-*value
Presence of varices	0.21	0.070–0.611	0.04
Brain natriuretic peptide (pg/mL)	1.01	1.003–1.017	0.04
Ejection fraction (%)	1.23	1.028–1.471	0.02

Factors were evaluated by sex, presence of varices, brain natriuretic peptide, and ejection fraction.

### Survival rates of LT candidates according to RVSP

**Fig 3:**
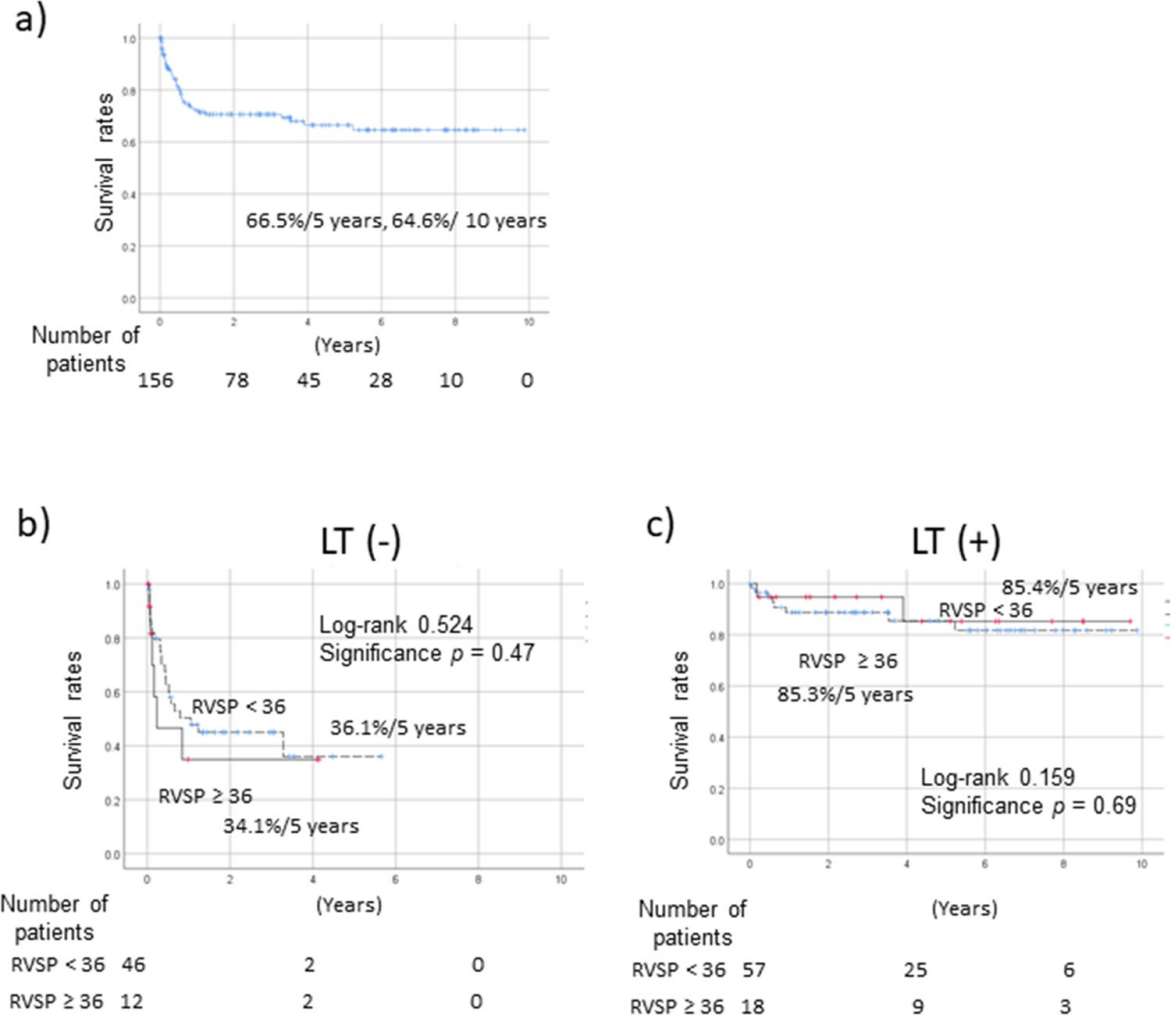
Mortality rates of cirrhotic LT candidates. (a) All patients and patients with RVSPs of < 36 and ≥ 36 mmHg (b) without LT and (c) with LT. The 5- and 10-year survival rates in cirrhotic LT candidates were 66.5% and 64.6%, respectively (a). About survival rate according to RVSP in LT and non-LT cases, in non-LT cases, the 5-year survival rates were 36.1% and 34.1% in those with RVSPs of < 36 and ≥ 36 mmHg, respectively (*p* = 0.47) (b). In LT cases, the 5-year survival rates were 85.4% and 85.3% in those with RVSPs of < 36 and ≥ 36 mmHg, respectively (*p* = 0.69) (c). LT, liver transplantation; RVSP, right ventricular systolic pressure.

The survival rates of cirrhotic LT candidates by Kaplan–Meier analysis are shown in Fig 3. The 5- and 10-year survival rates of cirrhotic LT candidates were 66.5% and 64.6%, respectively (Fig 3A). Prognosis was significantly poorer in patients without than with LT (*p* < 0.01). In a sub-analysis stratified by RVSP (< 36 vs. ≥ 36 mmHg) in LT and non-LT cases, the survival rates were similar. In non-LT cases, the 5-year survival rates were 36.1% and 34.1% in patients with RVSP values of < 36 and ≥ 36 mmHg, respectively (*p* = 0.47, Fig 3B). In LT cases, the respective rates were 85.4% and 85.3%, and did not differ by RVSP value (< 36 vs. ≥ 36 mmHg; *p* = 0.69, Fig 3C).

### Incidence of PoPH among LT candidates

Among 6 patients with an RVSP ≥ 50 mmHg, 3 (1.9%) were diagnosed with a PoPH complication by right heart catheterization at the time of considering LT (Table 3). PoPH cases had a significantly lower MELD score 10 than non-PoPH cases (17, *p* < 0.01). CTP score was not significantly different (PoPH 6 vs. non-PoPH 11, *p* = 0.13). Among three patients with PoPH, one of CTP class B-C died of progressive liver failure despite treatment for PoPH with tadalafil (a phosphodiesterase 5 [PDE5] blocker). The other two patients immediately started macitentan (ET receptor blocker), tadalafil, and/or selexipag (prostacyclin receptor agonist); as a result, mPAP was decreased and they were considered for LT. Among other 3 cases with an RVSP ≥ 50 mmHg, one case of Alagille syndrome was complicated by congenital aortic stenosis, pulmonary artery stenosis, spina bifida, and spinal canal stenosis. Congenital aortic and pulmonary artery stenosis increased the RVSP, resulting in pulmonary hypertension without symptom. This case was unlike PoPH; his EF was preserved and he underwent successful LT. In the other two patients with alcoholic liver disease and PBC, RVSP was increased by pleural effusion and ascites. After management with diuretic drugs, RVSP was decreased and LT was performed successfully.

**Table 3:** Characteristics of patients with PoPH.

Variable	Case 1	Case 2	Case 3
Age (years)	60	20	62
Sex	female	female	female
Primary liver disease	PBC	EHO	HCV, AIH, PBC
Other complications	Sjögren syndrome	Asthma	None
Child–Turcotte–Pugh score	10	6	6
MELD score	9	10	10
Esophageal/gastric varices or shunt	Esophageal varices, post EIS, EVL. Port-systemic shunt (Spleno-renal shunt)	Port-systemic shunt	Port-systemic shunt (spleno-renal shunt)
NYHA functional class	III-IV	III	III
WHO classification	III-IV	III	III
Brain natriuretic peptide (pg/mL)	733.7	90.8	142.4
Ejection fraction (%)	58	59	59
RVSP (mmHg)	106	77	122.4
Peak TR velocity (m/s)	4.9	4.1	5.3
Left atrium diameter (cm)	2.8	3.0	2.7
%/DLCO/VA (mL/min/mmHg/L)	2.59 (56.4%)	4.67 (93.4%)	3.3.1 (75.4%)
Hemodynamic variables			
Right atrial pressure (mmHg)	21	6	10
Mean pulmonary artery pressure (mmHg)	53	53	45
Pulmonary artery wedge pressure (mmHg)	14	8	11
Cardiac output (L.min−1)	3.13	5.2	5.5
Cardiac index (L.min−1)	2.0	3.4	3.5
Pulmonary vascular resistance (Wood units)	12.46	8.60	6.18
Mixed venous oxygen saturation (mmHg)	31.6 (53.7%)	45.4 (74.1%)	44.5 (78.3%)
Treatment for PoPH	Tadalafil	Macitentan	Macitentan
		Tadalafil	Tadalafil
			Selexipag
Outcome	Died due to liver cirrhosis	Considering LT after treatment of PoPH	Considering LT after treatment of PoPH

AIH, autoimmune hepatitis; %/DLCO/VA, diffusing capacity of lung for carbon monoxide/alveolar volume; EHO, extrahepatic portal vein obstruction; EIS, endoscopic injection sclerotherapy; EVL, endoscopic variceal ligation; HCV, hepatitis C virus; LT, liver transplantation; NYHA, New York Heart Association functional classification; TR, tricuspid regurgitation; PBC, primary biliary cholangitis; PoPH: portopulmonary hypertension; RVSP, right ventricular systolic pressure; WHO, World Health Organization.

## Discussion

The results revealed three cases (1.9%) of PoPH among LT candidates. The survival rates were not significantly different between patients with an RVSP < 36 and ≥ 36 mmHg suspected of having mild pulmonary hypertension, and the outcomes of LT were favorable. RVSP was increased in females and PBC patients, and weakly correlated with the BNP level.

LT candidates require comprehensive cardiopulmonary assessments, and all patients underwent screening echocardiography. Among the candidates for DDLT, 8.2% (101 of 1,235) had an RVSP > 50 mmHg according to echocardiography [7]. Among them, 90 had an mPAP > 25 mmHg and 66 had PAH, for a PoPH prevalence of 5.3% (66/1,235). Among the LT candidates, six cases (3.8%) had an RVSP of ≥ 50 mmHg and PoPH was observed in three cases.

Cardiac parameters including EF, peak TR velocity, and BNP were correlated with RVSP. Therefore, the measurement of serum BNP might be useful information for detecting PoPH. Yoshimaru et al. [29] reported that patients with PoPH had a significantly higher BNP level, which was predictive of asymptomatic PoPH; the optimal cutoff value was 29.1 pg/mL. In this study, median level of BNP was 39.1 pg/mL and it was 90.8 pg/mL in cases with RVSP ≥36. In terms of prognosis, there was no significant difference in the survival rate according to a BNP level (S2 Fig).

Patients with an RVSP ≥ 36 were more likely to be female, to have PBC and to have higher values for the cardiac parameters of BNP, EF, and mean peak TR velocity than those with an RVSP < 36. Kawut SM et al. [16] reported that female sex and autoimmune liver disease, especially PBC, were associated with pathogenesis of PoPH. Our RVSP data suggested their hypothesis. Esophageal varices was reported in 155 of 168 (92.3%) cases and in all patients with a mPAP > 20 [30]. In this study, esophageal/gastric varices were present in 108 cases (75.0%). The RVSP was lower in those cases compared to patients without variceal varices (*p* = 0.04). We speculate that RVSP can be increased by large shunts in the absence of esophageal/gastric varices; however, further analysis is needed.

Before the availability of PAH-specific therapies, LT was usually contraindicated because a large amount of blood flows into the right heart from the grafted liver, elevating the right cardiac pressure and PAP level [20, 22]. Advanced treatments for PAH have significantly improved survival [10, 22, 23]. The 5-year survival rates with PAH-specific therapy and LT, no therapy, and only medical therapy were 76%, 14%, and 45%, respectively [31]. The combination of PAH-specific therapy and LT yielded favorable outcomes. In our study, the 5-year survival rate was 86.8% in patients with an RVSP < 36 mmHg and 82.8% in those with an RVSP ≥ 36 mmHg. We performed LT in patients with a high RVSP, among whom 3 had an RVSP ≥ 50 and 16 an RVSP of 36–49. The mild to moderate RVSP cases underwent successful LT. In Japan, we must rely on LDLT due to a lack of available organs; moreover, the pressure on the portal vein might be increased by small-sized grafts. However, the surgical results were satisfactory in end-stage liver disease patients. Therefore, LT should be considered after or during ongoing treatment for PAH.

PoPH was diagnosed in three of our patients (1.9%, Table 3), a lower rate than in studies from Western countries [7]. Atsukawa et al. [30] reported that 1.1% of Japanese patients had PoPH, similar to our study. The PoPH cases were all female, and all had complicated autoimmune liver disease or extrahepatic portal vein obstruction (EHO). As the MELD score was poorly correlated with pulmonary hemodynamics [7], it was not related to the RVSP, similar to the CTP score. Actually, our PoPH cases showed a significantly lower MELD score and a tendency toward a decreased CTP score (one patient was CTP class B-C). These findings are comparable with a prior study [30]. We reported previously that two PoPH patients died due to severe liver and respiratory conditions, while two others survived for > 5–10 years after being treated with several drugs for PAH [32]. It was reported that the main causes of death in PoPH were complications of liver disease, particularly in patients with advanced cirrhosis [22]. In contrast, in patients with mild cirrhosis, the causes were PAH and hepatic or extrahepatic cancers. In the REVEAL registry of the United States, 5-year survival rates were significantly lower in patients with PoPH compared to idiopathic PAH (IPAH) (40% *vs*. 64%) [4]. Khaderi *et al*. [33] reported that, in patients with severe PoPH (7/488 cases), LT is feasible after reducing mPAP, and the survival rate after LT was 85.7% (median follow-up = 7.8 years). LT is indicated if mPAP can be controlled by vasodilators. Treatments for PAH might not be universally effective and the majority of PAH drugs are contraindication in liver cirrhosis with CTP class C. Therefore, it is important to diagnose PoPH at early stage of liver cirrhosis and LT should be considered in PoPH cases before progression to end-stage of liver cirrhosis. To diagnose PoPH at an early stage, screening by echocardiography should be performed for patients with liver cirrhosis or portal hypertension.

This study had several limitations. First, it was retrospective and involved a single center. Furthermore, not all patients underwent cardiac catheterization and we did not measure hepatic vein pressure. A prospective study of the outcomes of PoPH patients is needed.

In conclusion, the RVSP was increased in female and PBC patients. In the patients who underwent LT, survival was not associated with mild-moderate RVSP and the outcomes were favorable. PoPH cases had an RVSP of ≥ 50 mmHg; complications of PoPH should be screened by echocardiography in patients with portal hypertension being considered for LT.

## Supporting information

S1 Fig Frequency distribution of each parameter of liver and cardiac function. a) BNP, b) CTP score, c) MELD score, d) EF, e) RVSP, f) peak TR velocity, and g) LA diameter. BNP, brain natriuretic peptide; CTP, Child–Turcotte–Pugh; EF, ejection fraction; LA, left atrium; MELD, model for end-stage liver disease; RVSP, right ventricular systolic pressure; TR, tricuspid regurgitation.
(TIF)

S2 Fig About survival rate according to BNP level in LT and non-LT cases, there was no significant difference in the survival rate according to a BNP level. BNP, brain natriuretic peptide; LT, liver transplantation.
(TIF)
