# Supplementary figures and images for: Portopulmonary hypertension and the risk of high right ventricular systolic pressure in liver transplant candidates
Source: PLoS One. 2022 Apr 19;17(4):e0267125. doi: 10.1371/journal.pone.0267125 (PMC9017876; doi:10.1371/journal.pone.0267125)

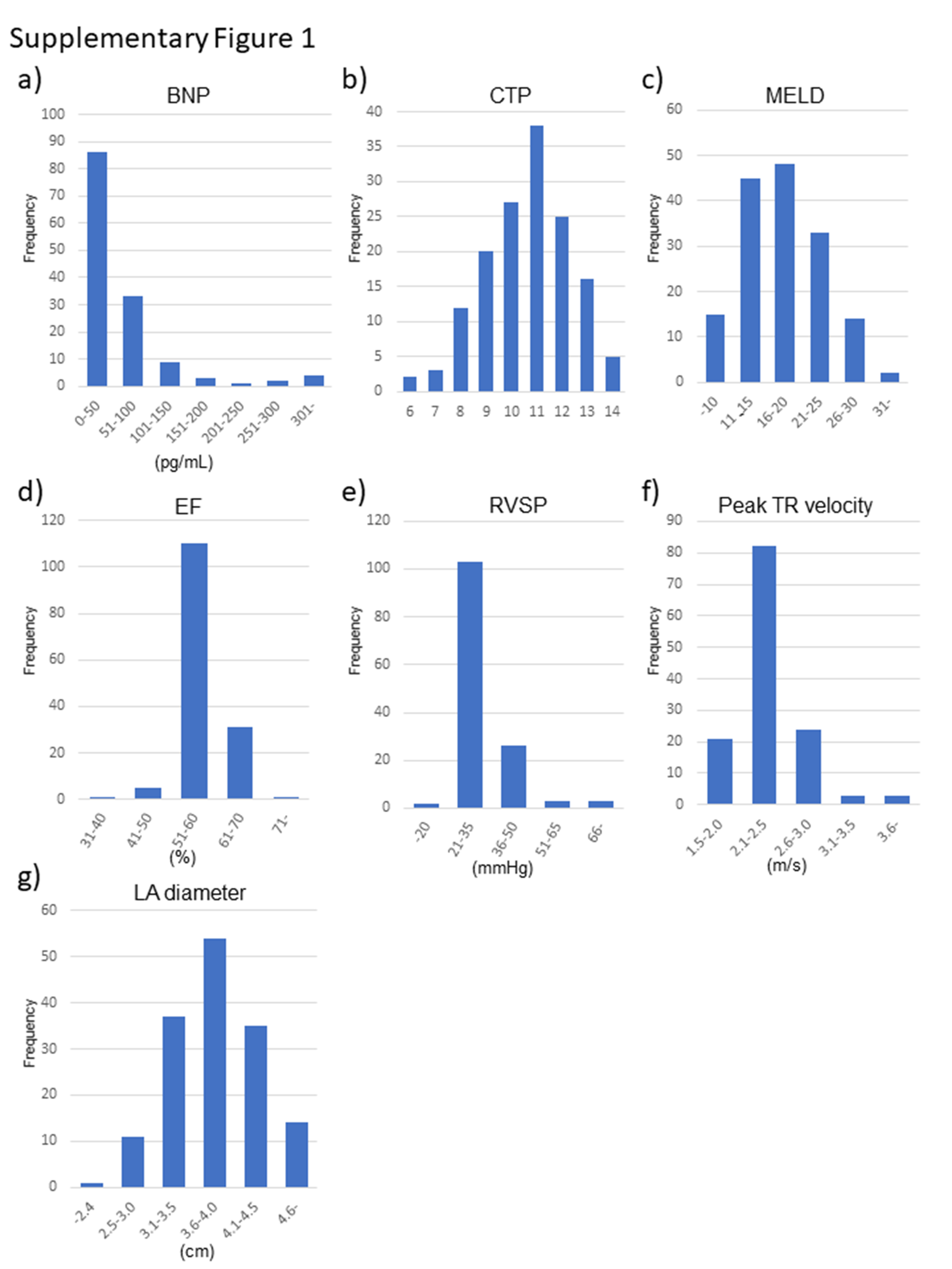

Supplement: S1 Fig — a) BNP, b) CTP score, c) MELD score, d) EF, e) RVSP, f) peak TR velocity, and g) LA diameter. BNP, brain natriuretic peptide; CTP, Child–Turcotte–Pugh; EF, ejection fraction; LA, left atrium; MELD, model for end-stage liver disease; RVSP, right ventricular systolic pressure; TR, tricuspid regurgitation. (TIF) [file pone.0267125.s001.tif]

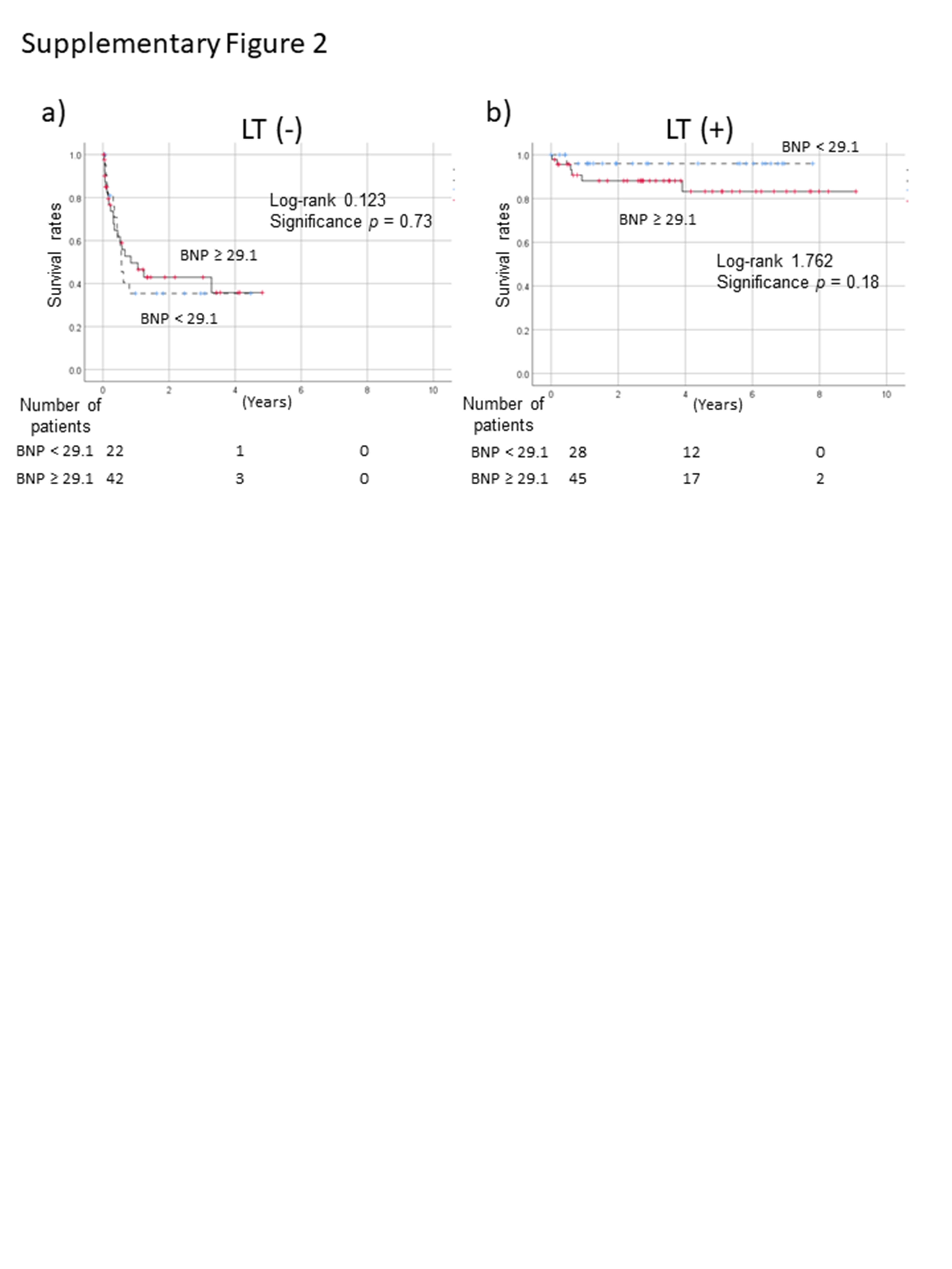

Supplement: S2 Fig — BNP, brain natriuretic peptide; LT, liver transplantation. (TIF) [file pone.0267125.s002.tif]
